# Probiotic Characteristics and Whole-Genome Profiling of *Lactiplantibacillus plantarum* V2 Isolated from Tibetan Plateau Traditional Yogurt

**DOI:** 10.3390/microorganisms14102222

**Published:** 2026-10-02

**Authors:** Lanxin Zhang, Man Chen, Zhaohai Wen, Junmei Li, Binghao Chen, Xianghong Ju, Yi Ma, Yanhong Yong, Xinbin Ma, Shuping Yan, Youquan Li

**Affiliations:** 1 Department of Veterinary Medicine, College of Coastal Agricultural Sciences, Guangdong Ocean University, Zhanjiang 524088, China; lanxin_zhang0203@163.com (L.Z.); chenman_may@163.com (M.C.);; 2 Marine Medical Research and Development Centre, Shenzhen Institute of Guangdong Ocean University, Shenzhen 518120, China

**Keywords:** *Lactiplantibacillus plantarum*, probiotic properties, evaluation, whole-genome sequencing, in vitro

## Abstract

*Lactiplantibacillus plantarum* V2 is a probiotic strain isolated from yogurt by Tibetan Plateau herdsmen in China. In this study, we systematically evaluated the probiotic potential and genome-wide features of *L. plantarum* V2. The cell-free supernatants (CFSs) of strain V2 exhibited broad-spectrum antimicrobial activity against at least 13 species of pathogenic bacteria. Moreover, *L. plantarum* V2 showed excellent gastrointestinal tract adaptability: it maintained a survival rate of 83.02 ± 1.64% at pH 3.0 an retained a survival rate of 20.34 ± 0.48% after 24 h exposure to 0.3% bile salts. Strain V2 adhered to Caco-2 cells with an efficiency of 1.83 ± 0.54%. It displayed non-hemolytic activity, was susceptible to clinically relevant broad-spectrum antibiotics, and harbored no transferable antimicrobial resistance genes. Whole-genome sequencing (WGS) analysis revealed that strain V2 possesses one circular chromosome and one linear plasmid, which collectively encode 3,041 coding sequences (CDSs), 16 rRNA genes, 71 tRNA genes, 12 CRISPR arrays, and a complete prophage region. Key functional genetic elements of strain V2 include five gene clusters encoding broad-spectrum antimicrobial peptides, six regions associated with environmental adaptability, 129 carbohydrate-active enzyme (CAZyme) genes, and one specialized metabolic cluster. Functional annotation further highlighted enriched pathways involved in transcriptional regulation, as well as the transport and metabolism of carbohydrates and amino acids. Collectively, our in vitro data demonstrate that *L. plantarum* V2 exhibits favorable acid and bile tolerance, potent bacteriostatic activity, and genomic safety, making it a promising candidate for applications in the livestock breeding and feed industries.

## 1. Introduction

Since the mid-20th century, antibiotics have been widely used in animal husbandry for disease prevention, treatment, and growth promotion [1]. However, their misuse and overuse have accelerated antibiotic-resistant microorganism (ARM) emergence, threatening zoonotic control, ecosystems, and public health [2]. After 2006, many countries banned antibiotic growth promoters (AGPs) in livestock feed per WHO’s AMR initiative, but intensive farming increases livestock disease risk, demanding antibiotic alternatives [3,4]. Probiotics (defined by ISAPP/WHO as “live microbes aiding hosts at adequate doses”) are promising—they modulate gut microbiota, inhibit pathogens, and reduce ARM excretion [5,6,7,8]. *Lactiplantibacillus plantarum* (formerly *Lactobacillus plantarum*), a safe Gram-positive anaerobe with EFSA’s QPS/FDA’s GRAS status, has broad antibacterial metabolites, aiding livestock silage and gut regulation to cut antibiotics [9,10,11,12,13,14].

Traditional probiotic screening methods are not only time-consuming but also fail to assess the genetic-level safety of probiotics [15]. In contrast, whole-genome sequencing (WGS) enables comprehensive characterization of probiotic strains, advancing the field of “probiotic genomics” to delineate host–microbe interaction mechanisms at the molecular level [16,17]. Despite the global identification of numerous probiotic strains with well-defined functions, there remains a scarcity of isolates exhibiting broad-spectrum bacteriostatic activity. Therefore, the successful screening of probiotic strains with broad-spectrum bacteriostatic properties, coupled with systematic in vitro probiotic characteristic evaluation and genetic-level analysis, holds significant scientific and practical value [18].

The extreme environmental conditions of the Qinghai–Tibet Plateau have driven the adaptive evolution of lactic acid bacteria (LAB) in locally fermented yogurt, endowing them with robust stress tolerance and enhanced functional traits. In our previous research, a total of 22 *L. plantarum* strains were successfully isolated. Preliminary unpublished results indicated that strain *L. plantarum* V2 exhibits excellent broad-spectrum antibacterial performance, with potent inhibitory effects on multiple dominant intestinal pathogenic bacteria, including *Escherichia coli*, *Salmonella typhimurium*, and *Mycoplasma* spp. Despite these findings, the comprehensive probiotic traits and genomic information of strain V2 have not been systematically explored and clarified. To fill this research gap, this study adopted conventional probiotic functional screening combined with whole-genome sequencing (WGS) technology to comprehensively analyze and evaluate the probiotic characteristics of *L. plantarum* V2. The obtained results are expected to provide a reliable theoretical basis and technical support for the popularization and application of this strain in the modern livestock breeding industry.

## 2. Materials and Methods

### 2.1. Isolation and Purification of LAB

A 10 mL aliquot of homemade yogurt, collected from herders in Menyuan (Qinghai–Tibet Plateau, China), was transferred into a centrifuge tube preloaded with 90 mL phosphate-buffered saline (PBS, pH 7.4; Servicebio, Wuhan, China). After 10-fold serial dilution, a 100 μL volume of each diluted suspension was uniformly spread onto de Man, Rogosa, and Sharpe (MRS; Beijing Luqiao Technology Co., Ltd., Beijing, China) agar plates supplemented with 1% (*w*/*v*) calcium carbonate. Lactic acid bacteria produce acidic metabolites during growth, which dissolve calcium carbonate in the medium and form a transparent zone; as such, the potential of a strain to be a lactic acid bacterium can be initially determined by the presence or absence of such a calcium-dissolving zone, and the strain’s acidogenic capacity can also be preliminarily estimated [19]. Plates were anaerobically incubated at 37 °C for 24 h. Colonies exhibiting morphological characteristics consistent with lactic acid bacteria (e.g., white/translucent, convex, and calcium-dissolving halos) were selected and transferred into 500 μL PBS-containing centrifuge tubes, followed by thorough mixing. An aliquot of the bacterial suspension was streaked onto fresh MRS solid medium plates, and this purification process was repeated for at least three generations to ensure clonality. Purified isolates were subjected to Gram staining to confirm cellular morphology.

### 2.2. Bacterial Strain and Growth Conditions

The V2 strain, *Lactobacillus casei* V5 and *Lacticaseibacillus rhamnosus* LGG were anaerobically cultured in MRS broth and on MRS agar plates, then preserved in MRS medium supplemented with 15% glycerol at −80 °C. For pathogenic bacterial strains, the following strains were used in the present study: *Bacillus cereus* ZJTP2, enterotoxigenic *Escherichia coli* (ETEC K88), *E. coli* ATCC 25922, *E. coli* O157:H7 ATCC 43888, *Staphylococcus aureus* ATCC 6538, *Proteus vulgaris* ATCC 29905, *Shigella boydii* ATCC 9207, *Yersinia enterocolitica* ATCC 23715, *Salmonella typhimurium* ATCC 14028, and *Pseudomonas aeruginosa* ATCC 27853. These pathogenic strains were aerobically cultured in nutrient broth and on nutrient agar (NA; Beijing Luqiao Technology Co., Ltd., Beijing, China) plates, then stored in glycerol-supplemented medium at −80 °C. In addition, seven *Mycoplasma* species—*Mycoplasma bovis*, *Mycoplasma capricolum* subsp. *capricolum*, *Mycoplasma ovipneumoniae*, *Mycoplasma mycoides* subsp. *capri*, *Mycoplasma agalactiae*, *Mycoplasma gallisepticum* and *Mycoplasma leachii*—were cultured in MTA medium at 37 °C under 5% CO_2_ and stored at −80 °C in glycerol-supplemented medium. All bacterial strains and *Mycoplasma* species used in this study were maintained in our laboratory.

### 2.3. Morphology and Molecular Identification

Molecular identification of the strains was conducted via PCR amplification and sequencing of the 16S rDNA gene. The PCR amplification protocol was as follows: initial denaturation at 95 °C for 5 min, followed by 35 cycles of denaturation at 94 °C for 45 s, annealing at 56 °C for 45 s, and extension at 72 °C for 50 s. The PCR products were analyzed by 1% agarose gel electrophoresis, revealing a single band of approximately 1.5 kb. Sequencing was conducted by Guangzhou Qingke Biotechnology Co., Ltd., (Guangzhou, China)and the resulting sequences were aligned against the NCBI database using the BLAST program (https://blast.ncbi.nlm.nih.gov/Blast.cgi; accessed on 15 May 2024)) for homology analysis. Morphological characterization of strain V2 included Gram staining and scanning electron microscopy (SEM). Gram staining was performed using a commercial kit (Solarbio Science & Technology Co., Ltd., Beijing, China) following the manufacturer’s protocols. For SEM sample preparation, strain V2 cells were fixed overnight in 2.5% glutaraldehyde at 4 °C. Samples were subjected to gradient dehydration with 30%, 50%, 70%, 80%, and 90% ethanol, followed by storage in anhydrous ethanol. Re-suspended cell droplets were pipetted onto silicon wafers and air-dried completely in a Petri dish. Dried samples were mounted on conductive adhesive, sputter-coated with gold at 10 mA using a Quorum SC7620 device (Quorum Technologies Ltd. Laughton, UK), and observed under a TESCAN MIRA LMS SEM (TESCAN GROUP, a.s. Brno, Czech Republic) at an accelerating voltage of 20 kV.

### 2.4. Hemolytic Activity

The hemolytic activities of *L. plantarum* V2 and *B. cereus* ZJTP2 were determined; *B. cereus* ZJTP2 was employed as the positive control in hemolysis tests. Bacterial cultures (including the test strain and control) were streaked on MRS agar plates supplemented with 5% (*v*/*v*) sterile sheep blood and incubated anaerobically at 37 °C for 24 h. Hemolytic activity was evaluated based on the following criteria: (i) α-hemolysis: formation of a greenish-yellow, semi-transparent zone around colonies; (ii) β-hemolysis: presence of a clear, colorless zone of complete hemolysis; and (iii) γ-hemolysis: no visible zone around colonies (non-hemolytic).

### 2.5. Antimicrobial Activity

In this study, the antibacterial activity of strain V2 against indicator strains was evaluated using the agar diffusion method. The antibacterial assay was performed with reference to the method described by Zhang et al. [20], with minor modifications. Enterotoxigenic *E. coli* (ETEC K88), *E. coli* ATCC 25922, *E. coli* O157:H7 ATCC 43888, *enterotoxigenic E. coli* (ETEC K88), *S. aureus* ATCC 6538, *P. vulgaris* ATCC 29905, *S. boydii* ATCC 9207, *Y. enterocolitica* ATCC 23715, *S. typhimurium* ATCC 14028, and *P. aeruginosa* ATCC 27853 were used for antibacterial experiments. *L. rhamnosus* LGG was used as a control. Following incubation at 37 °C for 24 h, the diameter of the inhibition zone was measured. The evaluation criteria are as follows: ≤9 mm indicates negative (−), 9–12 mm indicates weak positivity (+), 12–16 mm indicates strong positivity (++), and >16 mm indicates extremely strong positivity (+++).

Glycerol stocks of *M. bovis*, *M. capricolum* subsp. *capricolum*, *M. ovipneumoniae*, *M. leachii*, *M. mycoides* subsp. *capri, M. agalactiae,* and *M. gallisepticum* preserved in the laboratory were inoculated onto MTA plates and cultured in an incubator at 37 °C with 5% CO_2_. Colonies generally appeared on the plates after 3–5 days. Colony morphology was observed under an optical microscope, and single colonies were picked and transferred into 5 mL of MTA medium. The cultures were incubated at 37 °C until the medium color changed from red to yellow. Bacterial cells were collected by centrifugation at 12,000 r/min for 15 min in 1.5 mL tubes. The mycoplasma suspension was adjusted to a concentration of 1 × 10^6^ CFU/mL and evenly spread onto MTA plates using a sterile cotton swab. After standing at room temperature for 10–20 min, wells were punched in the agar (approximately 7 mm in diameter and 4.5 mm in depth) and sealed with 1% agar at the bottom. Then, 150 μL of cell-free supernatant from V2 was added into each well. The plates were kept at room temperature for 2 h before being transferred to an incubator at 37 °C with 5% CO_2_ for cultivation. Once clear inhibition zones appeared, the plates were removed, and the diameters of the inhibition zones were measured. *L. casei* V5 was used as the control strain.

### 2.6. Antibiotic Sensitivity Test

The antibiotic susceptibility profile of strain V2 was determined using the Kirby–Bauer disk diffusion method following Clinical and Laboratory Standards Institute (CLSI) guidelines [21]. Twelve antibiotics were tested (Hangzhou Microbial Reagent Co., Ltd., Hangzhou, China.) disks) containing the following concentrations: Ampicillin (10 µg), Kanamycin (30 µg), Gentamicin (10 µg), Chloramphenicol (30 µg), Streptomycin (10 µg), Tetracycline (30 µg), Nalidixic acid (30 µg), Amoxicillin (20 µg), Cefotaxime (30 µg), Erythromycin (15 µg), Vancomycin (30 µg), and Clindamycin (2 µg). MRS agar plates were inoculated with a standardized suspension of strain V2 (10^7^ CFU/mL) using the lawn culture technique. Antibiotic disks were aseptically placed on the inoculated medium, and plates were incubated aerobically at 37 °C for 24 h. *L. rhamnosus* LGG was used as a control. Inhibition-zone diameters were measured to the nearest millimeter. The results were categorized as resistant (R), intermediate (I), or susceptible (S). The specific evaluation criteria are listed in Supplementary Table S1.

### 2.7. Bile Salt and Gastric Acid Tolerance Test

As described by Lao et al. [22], bile salt and gastric acid tolerance tests were conducted on V2 strains with some modifications. The experiment was performed in duplicate three times. *L. rhamnosus* LGG was used as a control. After incubation of the samples under anaerobic conditions at 37 °C for 24 h, the absorbance of each mixture at 600 nm was measured to assess the growth of the strains. MRS-THIO broth without strain inoculation served as the control. The formula for calculating the survival rate is as follows:

(1) $$\mathrm{Survival}\;\mathrm{rate}(\%)\;=\;\left(\frac{\mathrm{OD}600\mathrm{nm}\;\mathrm{of}\;\mathrm{experimental}\;\mathrm{group}}{\mathrm{OD}600\mathrm{nm}\;\mathrm{of}\;\mathrm{control}\;\mathrm{group}}\right)\;\times \;100$$

Overnight cultures of the V2 strain were inoculated into artificial gastric juice (pH = 3; supplied by Dongguan Chuangfeng Automation Technology Co., Ltd., Dongguan, China) at a dilution ratio of 1:9 and incubated at 37 °C. Samples were collected at 0 and 3 h for plate counting. The survival rate of V2 in artificial gastric juice was calculated using the following formula:

(2) $$\mathrm{Survival}\;\mathrm{rate}(\%)\;=\;\left(\frac{3\;h\;\mathrm{live}\;\mathrm{bacteria}(\mathrm{CFU}/\mathrm{mL})}{0\;h\;\mathrm{live}\;\mathrm{bacteria}(\mathrm{CFU}/\mathrm{mL})}\right)\;\times \;100$$

### 2.8. Adhesion Ability to Caco-2 Cells

The adhesion assay was performed with slight modifications to the method described in reference [20]. The Caco-2 cell monolayer was washed twice with PBS, after which 1 mL of RPMI-1640 medium (Servicebio, Wuhan, China) containing 10^8^ CFU/mL of the V2 strain was added. The cells were then incubated at 37 °C under 5% CO_2_ for 2 h. After removing the culture medium, the cells were washed twice with PBS to ensure complete removal of unattached bacteria. Next, 1 mL of PBS containing 1% Triton X-100 was added to the cells, and the mixture was incubated at 37 °C for 10 min. The lysate was serially diluted with PBS, and the colonies were subsequently counted on MRS agar plates. The plates were incubated at 37 °C for 24–48 h, after which viable bacteria were counted. The experiment was performed in duplicate three times. *L. rhamnosus* LGG was used as a control. The adhesion rate was calculated using the following formula:

(3) $$\mathrm{Ad}\left(\%\right)=\left(\frac{V2\;\mathrm{concentration}\mathrm{after}2\;h}{V2\;\mathrm{initial}\mathrm{concentration}}\right)\times 100$$

### 2.9. Preliminary Evaluation of Antioxidant Properties

The DPPH free radical scavenging activity of V2 was measured using a commercial kit (Beijing Solarbio Science & Technology Co., Ltd., Beijing, China) following the manufacturer’s instructions. The assay is based on the decolorization of the deep-purple DPPH radical via a single-electron reduction. The V2 culture was adjusted to 1 × 10^8^ CFU/mL; 10 μL of this suspension was mixed with 190 μL of the DPPH working solution in a 96-well plate and incubated in the dark for 30 min. Absorbance was recorded at 515 nm. The blank group was prepared by replacing the sample with the extraction solvent, the control group by replacing the DPPH working solution with an equal volume of anhydrous ethanol, and the positive control group by replacing the sample with an equal volume of vitamin C solution. The scavenging rate (D%) was calculated as:

(4) $$D\left(\%\right)=\left(\frac{\mathrm{Ablank}-\left(\mathrm{Asample}-\mathrm{Acontrol}\right)}{\mathrm{Ablank}}\right)\times 100$$

The ABTS radical scavenging activity of V2 was determined using an ABTS radical scavenging activity assay kit (Beijing Solarbio Science & Technology Co., Ltd.) following the manufacturer’s instructions. The assay is based on the principle that ABTS is oxidized to generate a stable blue-green ABTS radical cation, which has maximum absorbance at 405 nm or 734 nm. Upon addition of the test sample, the antioxidant components present react with the ABTS radical cation, causing a decrease in absorbance at 405 nm. The V2 culture was adjusted to a concentration of 1 × 10^8^ CFU/mL, and 10 μL of this suspension was mixed with 190 μL of the mixed working solution in a 96-well plate. The reaction mixture was incubated in the dark for 6 min, and the absorbance was measured at 405 nm. In the blank group, the sample was replaced with distilled water; in the control group, the working solution was substituted with an equal volume of assay buffer; and in the positive control group, the sample was replaced with an equal volume of vitamin C solution. The ABTS radical scavenging rate (D%) was calculated as:

(5) $$D\left(\%\right)=\left(\frac{\mathrm{Ablank}-\left(\mathrm{Asample}-\mathrm{Acontrol}\right)}{\mathrm{Ablank}}\right)\times 100$$

### 2.10. Whole-Genome Sequencing of Strain V2

In this study, strain V2 was sent to Beijing Biomarker Technology Co., Ltd. (Beijing, China) for sequencing analysis using the Nanopore sequencer (Oxford Nanopore Technologies, Oxford, UK) [23]. The experimental procedures were performed strictly in accordance with the standard protocol provided by Oxford Nanopore Technologies (ONT), including sample quality assessment, library preparation, library quality evaluation, and sequencing. Low-quality and short fragments (<2000 bp) were filtered out, and the remaining high-quality reads were assembled using Canu v1.5. Subsequently, Racon v3.4.3 was used to correct the assembly results with third-generation sequencing reads [24]. Circularization and starting point adjustment were performed using Circlator v1.5.5. Finally, Pilon v1.22 was employed to further refine the genome assembly by leveraging second-generation sequencing data, yielding a highly accurate genome for downstream analysis. The average nucleotide identity (ANI) between the whole-genome sequences was calculated via FastANI software v1.33 [25]. Protein-coding genes (CDS) of the strain were identified via gene prediction using Prodigal v2.6.3 [26]. rRNA genes were predicted using Infernal v1.1.3, while tRNA genes were predicted using tRNAscan-SE v2.0 [27,28]. The prediction of other non-coding RNAs was primarily conducted using the Rfam database [29]. CRT v1.2 was used to identify CRISPR sequences across the entire genome [30]. IslandPath-DIMOB v0.2, PhiSpy v2.3, antiSMASH v8.0.1, gutSMASH v2.0.0, and PromPredict v1 were utilized to predict genomic islands, prophages, secondary metabolite gene clusters, primary metabolic gene clusters, and promoters, respectively [31,32,33,34,35]. Gene function annotation was performed using the Non-Redundant Protein Database (Nr), Gene Ontology (GO), Kyoto Encyclopedia of Genes and Genomes (KEGG), eggNOG, Pfam, SwissProt, and TrEMBL databases [36,37,38,39,40,41]. Additionally, carbohydrate-active enzymes were predicted using the Carbohydrate-Active enZYmes database (CAZy), and virulence factors were identified using the Virulence Factor Database (VFDB) [42,43]. PHI-base was used to predict genes involved in pathogen–host interactions in the genome [44]. Pathogenicity and drug resistance analyses were conducted using the Comprehensive Antibiotic Resistance Database (CARD), ResFinder and blastKOALA [45,46,47].

### 2.11. Statistical Analysis

All experiments were performed in triplicate to ensure data reliability, and the results are presented as mean ± standard error (SE). All the statistical analyses were performed via IBM SPSS Statistics 26 software.

## 3. Results

### 3.1. Identification and Morphological Observation of the V2 Strain

**Figure 1:**
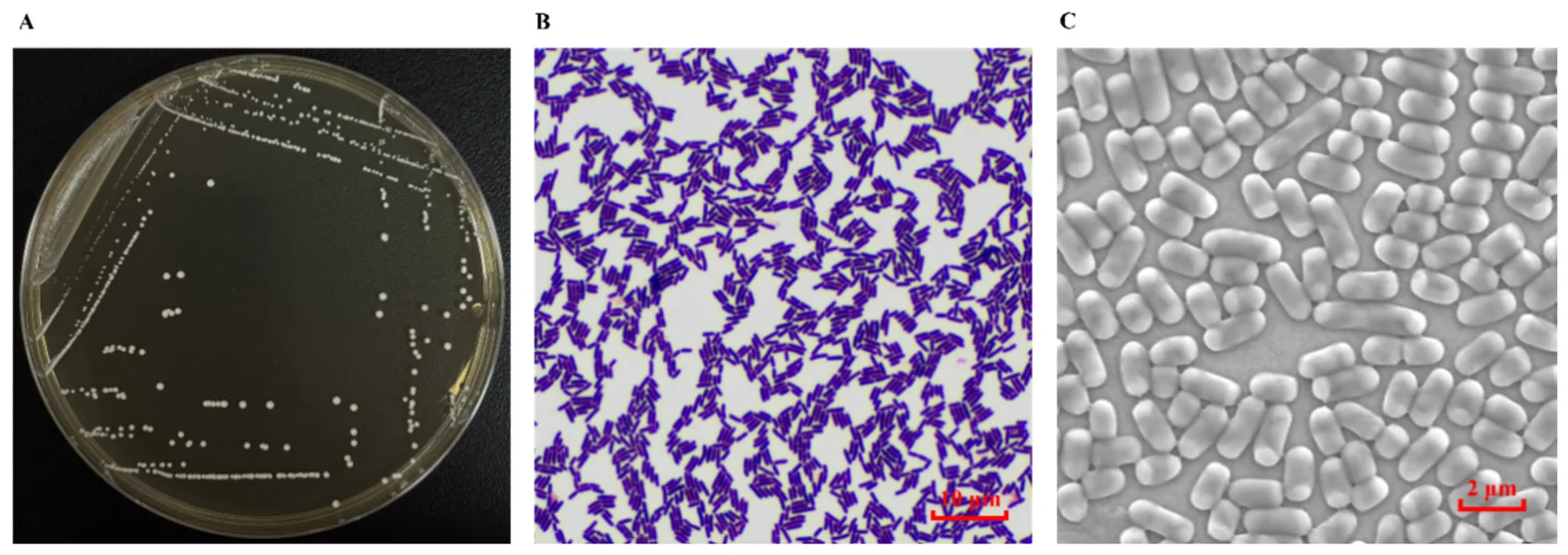
Morphological characterization of strain V2. (**A**) Colonial morphology. (**B**) Gram staining (1000×). (**C**) Scanning electron microscopy.

A total of 22 strains of *L. plantarum* were isolated from yogurt produced in Qinghai Province. Based on hemolytic activity, antibacterial activity, and other probiotic properties, only strain V2 was ultimately selected for subsequent research. In this study, as shown in Figure 1A, the V2 colonies displayed a circular, raised, and smooth morphology with a slightly milky white appearance, which is consistent with the typical morphological characteristics of lactic acid bacteria. Gram staining showed Gram-positive bacilli with a characteristic blue-purple staining pattern (Figure 1B). Scanning electron microscopy analysis revealed that strain V2 had a smooth, rod-shaped morphology (Figure 1C).

### 3.2. Hemolytic Activity Analysis

**Figure 2:**
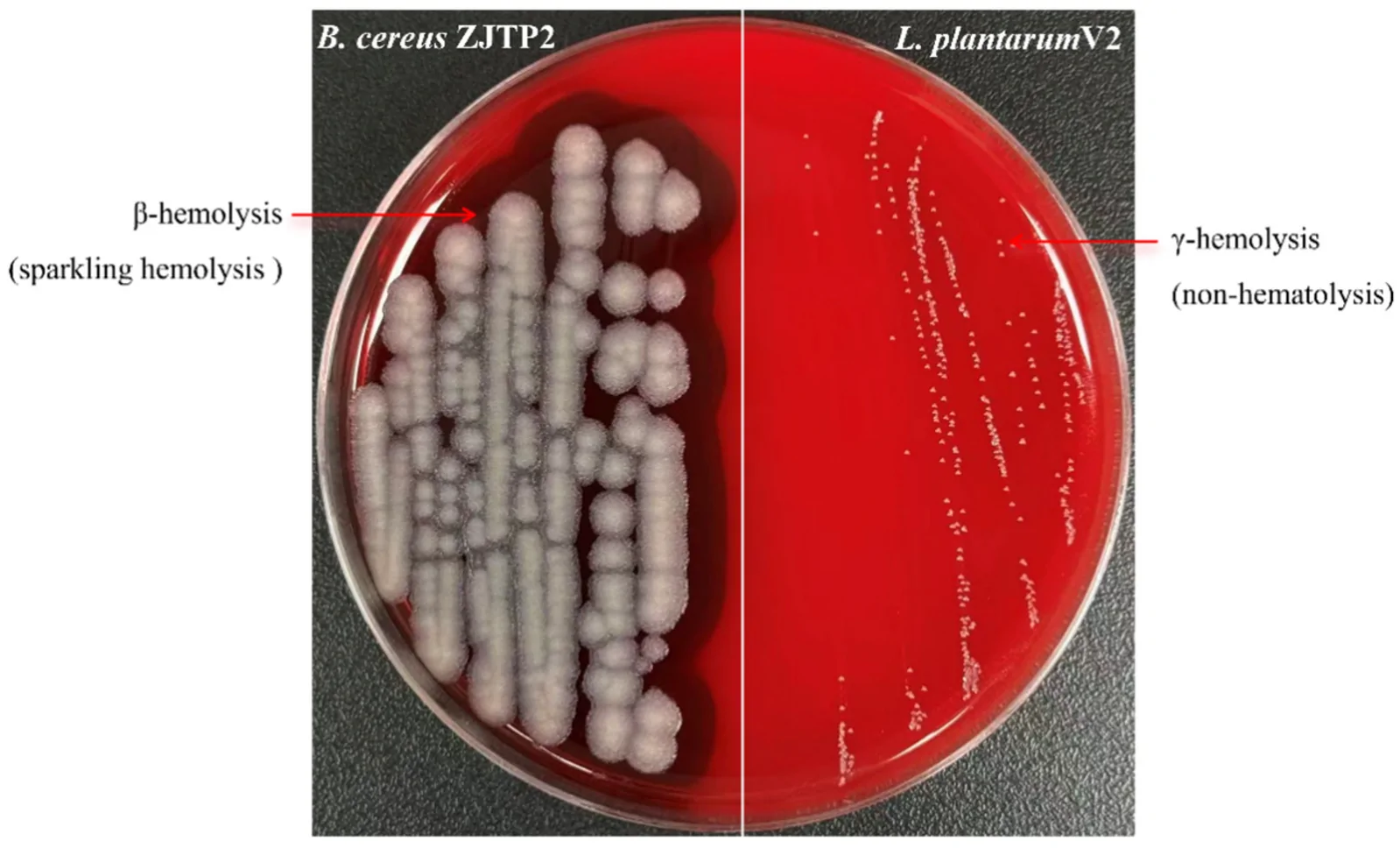
Hemolytic results of strain V2. Hemolysis results of the control strain (*B. cereus* ZJTP2) are shown on the left and γ-hemolysis (non-hematolysis) of strain V2 on the right.

The hemolytic results are shown in Figure 2. Enterotoxigenic *B. cereus* ZJTP2 produced a distinct, transparent hemolytic zone (β-hemolysis), whereas strain V2 showed no hemolytic zone (γ-hemolysis).

### 3.3. Antibacterial Activity Analysis

Table 1 shows the antibacterial activity of strain V2 against nine pathogenic bacterial strains. Strain V2 displayed inhibitory activity against all nine pathogenic strains. The strongest inhibitory effect was observed against *Y. enterocolitica*, with an inhibition-zone diameter of 24.13 ± 0.50 mm. The second-highest inhibitory activity was detected against *S. boydii*, with an inhibition-zone diameter of 23.90 ± 0.47 mm. In addition, strain V2 exerted inhibitory effects against four tested *Mycoplasma* species; the maximum inhibition was found for *M. leachii*, yielding an inhibition-zone diameter of 16.59 ± 0.23 mm (Table 2).

### 3.4. Antibiotic Resistance Analysis

The susceptibility of strain V2 to twelve antibiotics was tested, and the results are shown in Table 3. Strain V2 was sensitive to three antibiotics, with high susceptibility to Ampicillin and Amoxicillin, both of which produced inhibition zones exceeding 21 mm in diameter.

### 3.5. Tolerance to Bile Salts and Gastric Acid

Gastric acid typically exhibits a pH of approximately 3, while the concentration of bile salts in digestive fluids ranges from 0.03% to 0.3%. Under physiological conditions, food resides in this digestive environment for roughly 1 to 3 h [48]. Accordingly, artificial gastric juice (adjusted to pH 3) and 0.3% bile salt solution were employed to assess the tolerance of strain V2. The results showed that the 3 h survival rate of strain V2 in artificial gastric juice (pH 3) was 83.02 ± 1.64%, and its 24 h survival rate in a 0.3% bile salt environment was 20.34 ± 0.48%. In the control group, the survival rate of *L. rhamnosus* LGG in artificial gastric juice (pH 3) for 3 h was 72.57 ± 0.26%, and the survival rate in the 0.3% bile salt environment for 24 h was 18.56 ± 0.37%.

### 3.6. Adhesivity

Probiotics exert their beneficial effects more efficiently through adhesion to intestinal epithelial cells or colonization of the mucus layer [49]. By competing with pathogens for essential nutrients and adhesion sites, probiotics can inhibit pathogenic colonization via competitive exclusion. The adhesion assay demonstrated that the adhesion rate of strain V2 and *L. rhamnosus* LGG to Caco-2 cells was 1.83 ± 0.54% and 8.02 ± 0.40%, respectively.

### 3.7. Antioxidant Capacity Analysis

The strain exhibited DPPH and ABTS radical scavenging rates of 59.91 ± 0.65% and 80.96 ± 1.24%, respectively.

### 3.8. Genomic Features of L. plantarum V2

**Figure 3:**
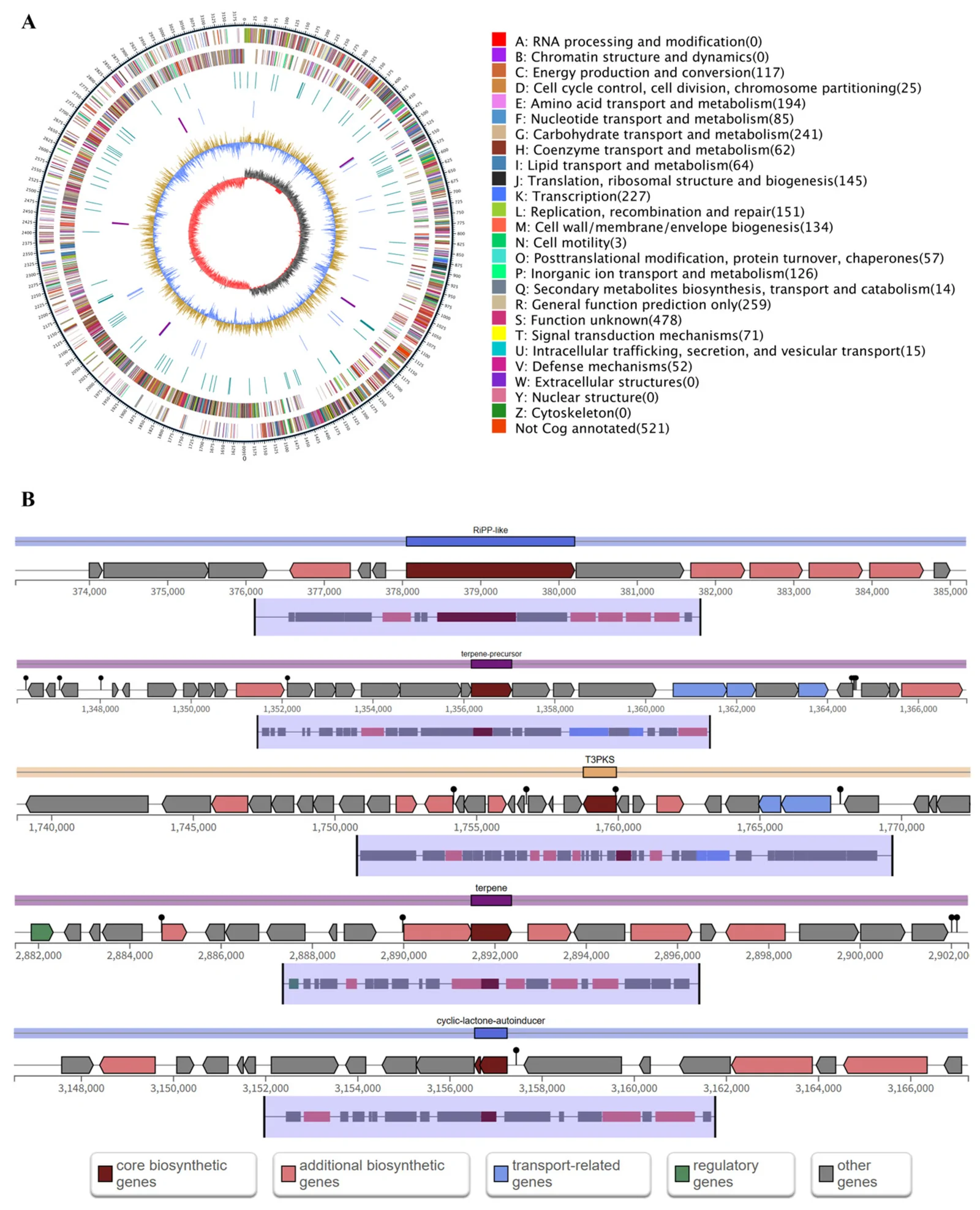
Circular genome map of strain V2 and predicted biosynthetic gene clusters for secondary metabolites. (**A**) The circular map of the assembled genome of strain V2. (**B**) The prediction results of the secondary metabolite gene clusters of strain V2 predicted by antiSMASH v8.0.1.

The genome of *L. plantarum* V2 has a total length of 3,261,808 bp, consisting of a circular chromosome (3,197,739 bp) and a linear plasmid (64,069 bp), with an average GC content of 44.48% (Figure 3A). It contains 3,041 coding sequences (CDSs). Additionally, 16 rRNA genes were identified, including five 23S rRNA, six 5S rRNA, and five 16S rRNA genes, along with 71 tRNA genes. The major genomic features of strain V2 are summarized in Table 4. The whole-genome sequence of *L. plantarum* V2 has been deposited in the NCBI database under the accession numbers GenBank: CP180259–CP180260.

The results of ANI analysis for the 20 strains are shown in Figure S1. An ANI value greater than 95% indicates that two bacterial strains belong to the same species. The ANI values between V2 and the genomes of the other 19 bacterial strains ranged from 76.86% to 99.49%, indicating differences in genomic similarity among these organisms. Among them, the ANI values of V2 with the five *L. plantarum* strains included in the analysis were all greater than 98%, and the ANI values for *L. plantarum* cqf-43 and WCFS-1 (which have been confirmed to have multiple probiotic functions) were greater than 99%. The NCBI accession numbers and names of the 19 bacterial strains are listed in Supplementary Table S5.

Secondary metabolites produced by bacteria are crucial sources of antibacterial agents and various bioactive compounds. Using antiSMASH v8.0.1, a total of five gene clusters were identified on the chromosome (Figure 3B), classified as RiPP-like, terpene-precursor, T3PKS, terpene, and cyclic-lactone-autoinducer types, respectively. The first region spans from 373,057 bp to 385,207 bp, within which the synthesis genes of two-peptide bacteriocin plantaricin EF—ACMATP_01730 and ACMATP_01735—were identified.

#### 3.8.1. Genome Annotation

To characterize the genomic features of strain V2, genome annotation was performed using seven functional databases, including Nr, GO, KEGG, and eggNOG. Table 5 summarizes the number of genes annotated across these general databases.

**Figure 4:**
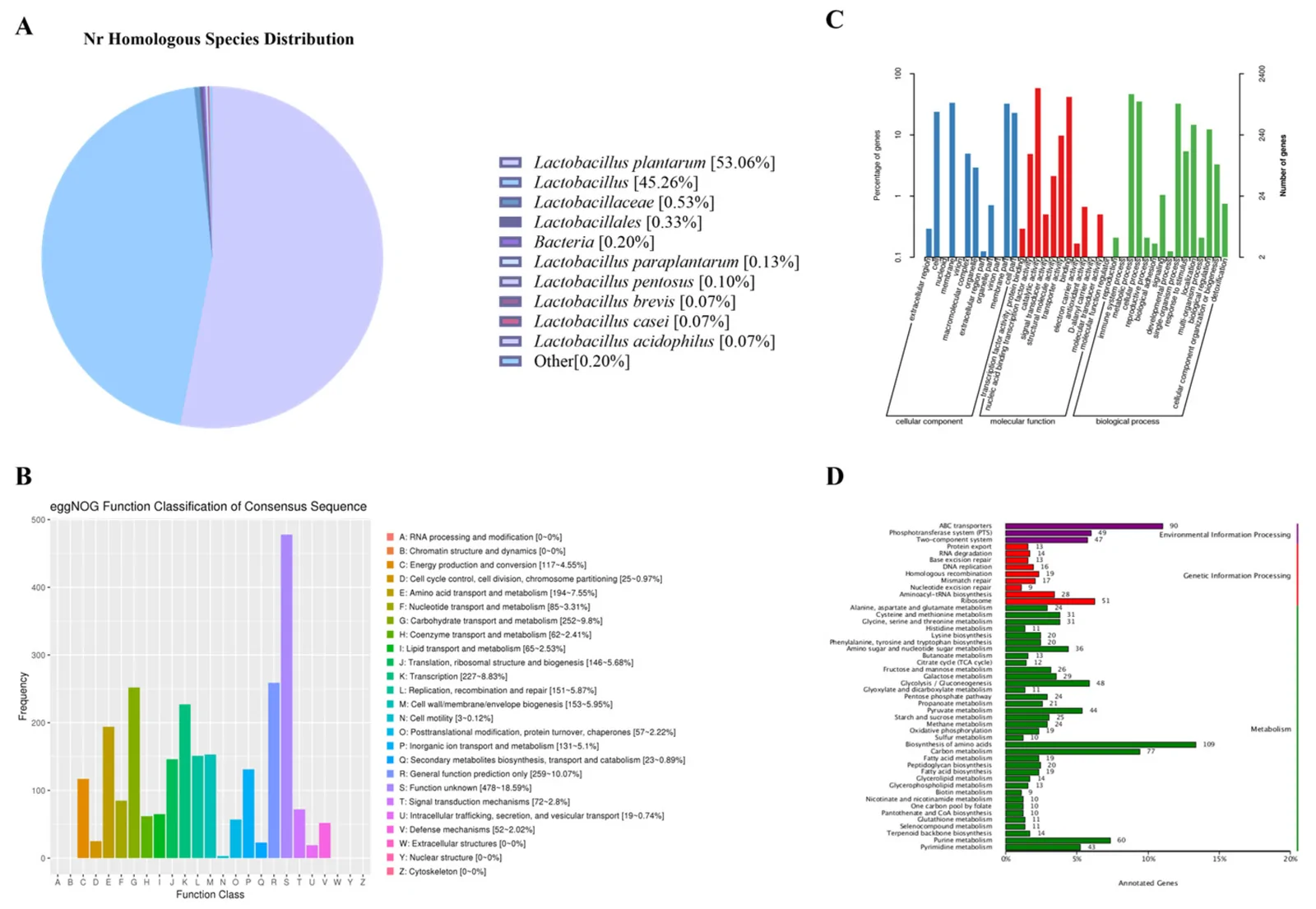
Universal functional annotation map of the whole genome of strain V2. (**A**) Species distribution map of sequences aligned to the Nr database, illustrating the taxonomic composition of matched sequences. Different colors represent distinct species. (**B**) eggNOG functional gene classification chart, displaying the distribution of gene functions across different eggNOG categories. The x-axis represents various eggNOG functional classifications, and the y-axis indicates the relative proportion of corresponding functional gene counts. (**C**) GO functional annotation classification chart, illustrating the distribution and enrichment of genes across secondary functional categories of Gene Ontology (GO). The horizontal axis represents different GO classifications; the left vertical axis indicates the percentage of gene counts, and the right vertical axis displays the absolute number of genes. This chart reflects the relative importance of each secondary functional category within the overall gene set. (**D**) KEGG annotation classification chart, illustrating the distribution of gene functions across KEGG secondary categories. The vertical axis represents functional classifications, and the horizontal axis indicates the relative percentage of each category.

Figure 4A shows the annotation results of protein sequences of strain V2 in the Non-Redundant Protein Database (Nr database). Specifically, 1612 proteins were annotated as *L. plantarum*, accounting for 53.06% of the predicted proteins, and 1375 proteins were annotated at the genus level as *Lactobacillus*, representing 45.26% of the predicted proteins.

The eggNOG annotation analysis (Figure 4B) revealed that the V2 genome contains a substantial number of protein-coding genes with unknown functions (Class S, *n* = 478), suggesting that this strain may encode diverse and potentially unique proteins. The number of genes associated with “General function prediction only” (Class R, *n* = 259) in the V2 genome is particularly prominent. Additionally, the genome harbors functional genes involved in carbohydrate transport and metabolism (Class G, *n* = 252), transcription (Class K, *n* = 227), and amino acid transport and metabolism (Class E, *n* = 197). Based on the Gene Ontology (GO) database (Figure 4C), gene functions are categorized into three primary domains: cellular components, molecular functions, and biological processes. Analysis reveals that in the cellular component category, the majority of genes are associated with cells and membranes (54.00%); in the molecular function category, most genes are linked to catalytic activity (48.83%); and in the biological process category, metabolic processes account for the largest proportion (53.51%).

The Kyoto Encyclopedia of Genes and Genomes (KEGG) pathway annotation results are shown in Figure 4D. The coding sequences (CDSs) of strain V2 are predominantly enriched in three secondary KEGG pathway categories: Biosynthesis of Amino Acids, ABC Transporters, and Carbon Metabolism, with the highest number of genes found in the Biosynthesis of Amino Acids pathway.

#### 3.8.2. Exclusive Database Annotations

To explore the potential of strain V2 for carbohydrate-active enzyme production and secreted protein synthesis, as well as its putative functional traits, genome annotation was performed using eight functional databases, including the Carbohydrate-Active enZYmes database (CAZy), Comprehensive Antibiotic Resistance Database (CARD), and Virulence Factors of Bacterial Pathogens database (VFDB). Table 6 summarizes the genome annotation results of strain V2 obtained from these specialized databases.

**Figure 5:**
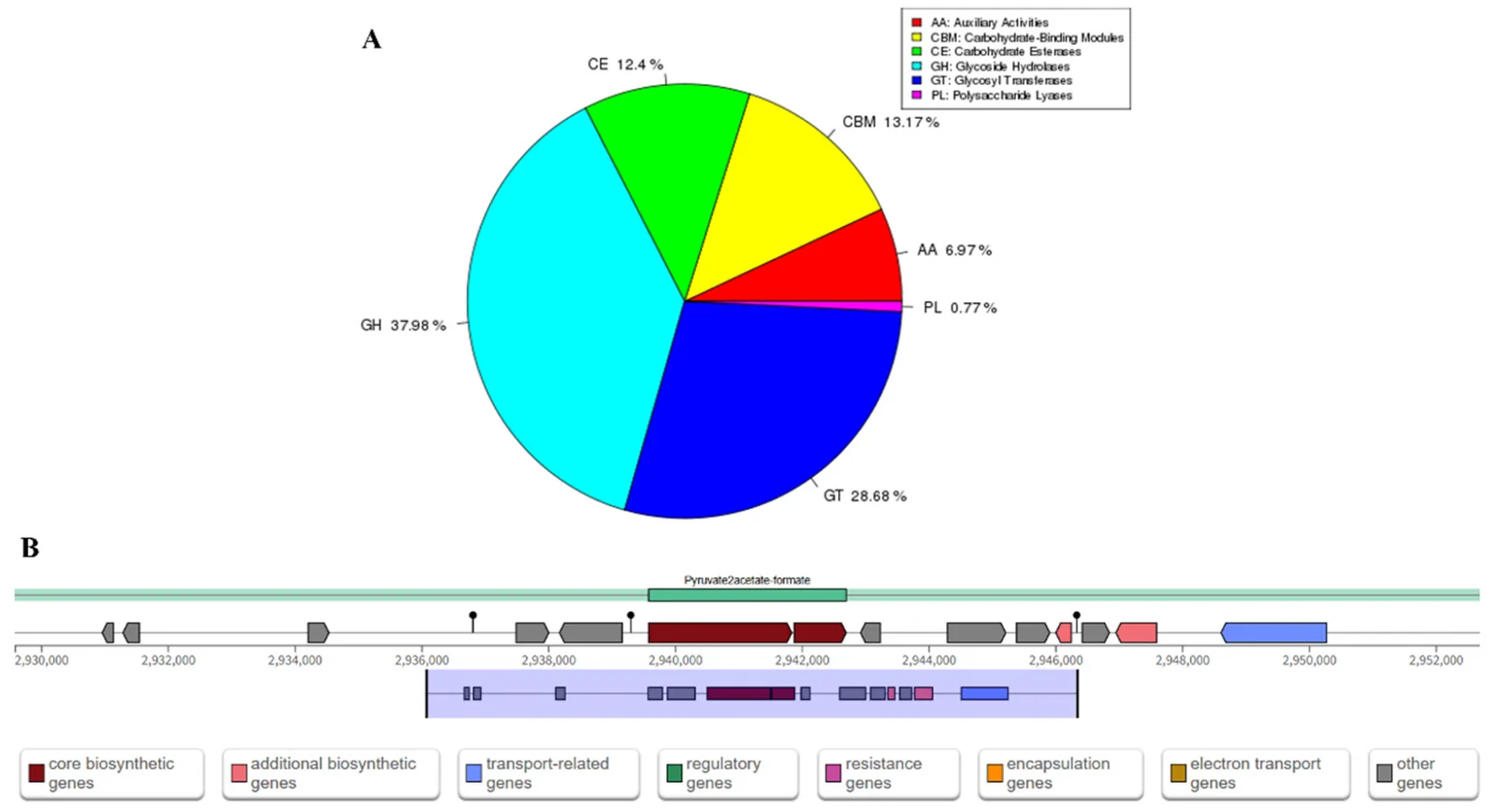
CAZy database annotation of strain V2 and gutSMASH v2.0.0 annotation results. (**A**) Distribution ratio graph of carbohydrate-active enzymes. Different functional categories are represented by different colors. (**B**) SCFA metabolic gene clusters annotated by gutSMASH v2.0.0.

Through analysis of the Carbohydrate-Active enZYmes database (CAZy), 129 genes in the genome of strain V2 were identified as CAZy enzymes, which were classified into six categories (Figure 5A). Among these, glycoside hydrolases (GHs) accounted for 37.98% of the total annotated genes, followed by glycosyltransferases (GTs) at 28.68%, non-catalytic carbohydrate-binding modules (CBMs) at 13.17%, carbohydrate esterases (CEs) at 12.40%, and auxiliary activities (AAs) at 6.97%. Polysaccharide lyases (PLs) represented only 0.77% of the total annotated genes. Genome annotation using gutSMASH v2.0.0 revealed a short-chain fatty acid metabolic gene cluster in the genome of strain V2 (Figure 5B).

Using the Comprehensive Antibiotic Research Database (CARD), the resistance gene *poxtA* was annotated. This gene encodes a ribosome protection protein belonging to the ABC-F subfamily, which may reduce microbial susceptibility to antibiotics or confer resistance to Oxazolidinones, Chloramphenicol, and Tetracyclines. Notably, no acquired resistance genes were predicted through ResFinder 4.5.0. Fourteen antimicrobial resistance genes were identified by BlastKOALA. These were mainly bacterial multidrug resistance genes that may lead to resistance to antibiotics such as Tetracycline. A total of 108 related genes were annotated via the VFDB, all of which exhibited sequence similarities below 50%. These genes are primarily associated with the synthesis of adhesion proteins, transport proteins, and biologically functional enzymes. Notably, annotation using the VFDB revealed that strain V2 possesses potential bile salt hydrolase (BSH) activity. The 478 genes annotated through PHI-base exhibit relatively low sequence similarity, with the majority categorized by PHI-base as “Reduced virulence” or “Loss of pathogenicity.”

## 4. Discussion

In vitro probiotic characterization and whole-genome analysis are critical technical methodologies for determining the suitability of candidate probiotic strains for industrial development and practical application. Among these, anti-pathogenic activity and safety are regarded as the core evaluation criteria for lactic acid bacteria (LAB) to be considered as probiotic candidates [50,51]. In this study, *L. plantarum* V2 is active against at least thirteen common pathogens, including intestinal pathogenic bacteria and respiratory pathogenic bacteria. This highlights the potential application of strain V2 in the prevention and treatment of diseases such as pneumonia caused by mycoplasmosis and diarrhea induced by intestinal pathogenic bacteria in clinical settings. Current evidence indicates that *L. rhamnosus* GG can modulate respiratory immune responses and alleviate *Mycoplasma pneumoniae*-induced pneumonia in mouse models when administered intranasally [52]. García-Galán et al. [53] demonstrated that supplementing with *Lactobacillus* at concentrations above 10^8^ CFU/mL can reduce the survival rate of *M. bovis* in bovine cervical mucus and lower the risk of *M. bovis* transmission during artificial insemination. In addition, the antibacterial activity of lactic acid bacteria is primarily attributed to metabolic byproducts such as organic acids, hydrogen peroxide, and bacteriocins [54,55,56]. Furthermore, gastrointestinal-specific enzymes, adverse pH conditions, and mild heat shock in the intestinal microenvironment can significantly enhance the antimicrobial potency of these compounds [57,58]. These factors suggest that oral administration may yield a more pronounced antibacterial effect. *L. plantarum* V2 exhibits no hemolytic activity and lacks virulence factors directly involved in host interaction, yet it harbors genes associated with bacterial adhesion, colonization, and chemical resistance according to genome-wide analysis. These genes may contribute to the robust stress resistance of strain V2.

Tolerance to gastric acid and high concentrations of bile salts is critical for the survival and functional efficacy of probiotics in the host [59]. To withstand these harsh conditions, probiotics must either possess intrinsic resistance mechanisms to acid and bile stress or develop alternative metabolic strategies to ensure intestinal colonization [60]. Generally speaking, the stress resistance of probiotics is strain-specific [8,61]. In this study, strain V2 could survive and proliferate in artificial gastric fluid and bile salts and exhibited potential bile salt hydrolase (BSH) activity in vitro and in genome-wide analyses. BSH facilitates the incorporation of cholesterol or bile components into the bacterial membrane, modifying membrane fluidity and surface charge, which in turn alters susceptibility to α-defensins and other host-derived antimicrobial molecules [62]. Probiotics utilize BSH to enhance their survival and stability in the intestinal environment [63]. Furthermore, probiotics can exert beneficial effects such as cholesterol reduction via BSH-mediated bile acid metabolism [64]. Accordingly, BSH in probiotics remains a focus of ongoing research. While probiotic efficacy does not exclusively depend on long-term host colonization, strong adhesive properties are critical for establishing microecological competitive advantages [65]. The adhesion ability of probiotics enhances their ability to interact with the host, which can prolong their residence time in the gastrointestinal tract, thereby increasing the time to exert probiotic effects [66]. Although strain V2 does not display strong adhesion capacity, this does not preclude it from exerting probiotic functions. Seo et al. [67] reported that *L. plantarum* RP12, a grain-derived isolate, showed a Caco-2 cell adhesion rate of 1.67%, yet it was able to inhibit adipogenesis in adipocytes, supporting its potential as a probiotic candidate for functional nutraceutical development. Similarly, Ramos et al. [68] found that *L. plantarum* PV018 exhibited only a low Caco-2 adhesion rate of 1.3 ± 0.2%. Despite this relatively weak adhesion, PV018 was regarded as a promising probiotic strain owing to its robust tolerance to bile salts and acidic conditions, together with favorable antibacterial activity. Studies have shown that the mechanisms of action of certain *Saccharomyces boulardii* are not entirely adhesion-dependent [69]. Some probiotics act as transient residents in the gastrointestinal tract rather than permanently colonizing the gut. Their beneficial effects may be mediated via the production of SCFAs, bacteriocins and other bioactive metabolites during intestinal transit; these compounds modulate host immune responses and contribute to overall probiotic activity [70,71]. Previous studies have demonstrated that SCFAs, including butyrate and acetate, preserve intestinal barrier integrity through the activation of AMPK and TLR4 signaling [72]. For example, *Bifidobacterium longum* protects gut epithelial cells from infection by *E. coli* O157 via acetate secretion [73]. Lu et al. [74] reported that *L. plantarum* A5 alleviates high-fat-diet-induced hyperlipidemia by remodeling gut microbiota to boost short-chain fatty acid biosynthesis. Although the mechanisms underlying bacteriocin activity remain incompletely characterized, De Giani et al. [75] revealed that bacteriocins eliminate target bacteria by permeabilizing cell membranes or binding to specific membrane proteins known as bacteriocin receptors. This peptide–receptor interaction induces membrane leakage and subsequent bacterial cell death. Corr et al. [76] further demonstrated that bacteriocin Abp118, produced by *L. salivarius* UCC118, confers robust protection in mice against infection with the invasive foodborne pathogen *Listeria monocytogenes*. Consistent with these reported mechanisms, strain V2 harbors short-chain fatty acid (SCFA) metabolic pathways and bacteriocin gene clusters, which may represent the key mechanisms underlying its probiotic efficacy.

Oxidative stress can induce DNA hydroxylation, protein denaturation, and lipid peroxidation, ultimately compromising cell viability. In this study, the V2 strain exhibited DPPH and ABTS radical scavenging rates of 59.91 ± 1.12% and 80.96 ± 2.15%, respectively, indicating its favorable antioxidant capacity. Previous studies have reported that *L.plantarum* 200655 showed DPPH and ABTS radical scavenging rates of 30.51% and 38.13%, respectively; *L. plantarum* GP displayed a DPPH scavenging activity of 77 ± 1.2%; and *Pediococcus acidilactici* CLP03 exhibited scavenging rates of 53.55% against DPPH radicals and 54.81% against ABTS radicals [77,78,79]. Notably, V2 demonstrated high scavenging efficiency against both radicals, suggesting its broad-spectrum antioxidant potential. It is well known that certain probiotic strains can produce various antioxidative substances, including superoxide dismutase, glutathione peroxidase, and vitamins; it is reasonable to speculate that V2 may also synthesize such molecules, thereby enhancing its overall radical-scavenging capacity.

Antibiotics are widely used in livestock; however, their overuse drives the emergence of antimicrobial resistance, the dissemination of antibiotic resistance genes (ARGs) through the food chain (posing threats to public health), and disruption of the intestinal microbiota, accompanied by substantial economic losses. Probiotics represent promising alternatives, and assessment of the antibiotic susceptibility of candidate strains is therefore critical for mitigating ARG dissemination. In this study, the drug resistance of the V2 strain was similar to that of previously reported strains [80,81]. The development of antibiotic resistance in bacterial strains may be influenced by the specific types of antibiotics used during rearing. Prolonged exposure to single or combined antibiotics can promote the emergence of resistant strains and facilitate horizontal transfer of resistance genes [82]. Safety assessment of probiotic strains should consider not only the presence of antimicrobial resistance (AMR) genes and virulence-associated genes but also their genetic backgrounds and potential mobility. In particular, acquired AMR genes carried on plasmids or other mobile genetic elements exhibit elevated potential for horizontal gene transfer, underscoring the necessity to evaluate their risk of transfer [83,84]. Whole-genome analysis identified the *poxtA* gene in strain V2. Notably, *poxtA* is commonly found in lactic acid bacteria [85]. This gene encodes a ribosome protection protein belonging to the ABC-F family, which can mediate resistance to Oxazolidinones, Chloramphenicols, and Tetracyclines [86]. The presence of the *poxtA* gene does not necessarily confer antibiotic resistance, as its phenotypic expression depends on its expression level, which may be influenced by plasmid copy number, chromosomal copy number, and genetic background. Strains E2918LS and E3995LS carried nine copies of the *poxtA* gene per chromosome but remained susceptible to linezolid. In contrast, strain E6786LR carried the lowest number of *poxtA* gene copies (one copy per chromosome) but exhibited the highest PoxtA protein level, with the linezolid MIC exceeding the maximum detectable concentration (>256 µg/mL) [87,88]. Although strain V2 harbors the *poxtA* gene, the antibiotic susceptibility test showed that V2 was not resistant to Chloramphenicol or Tetracycline, which may be related to the low expression level of the *poxtA* gene, which is insufficient to confer resistance. Studies indicate that *PoxtA* mediates resistance to Phenicols and Oxazolidinones but does not confer resistance to Tetracycline-class antibiotics [86]. Since *poxtA* is chromosomally encoded in strain V2, it poses no risk of horizontal ARG transfer. The CARD did not identify other resistance genes of strain V2, probably because the database is more suitable for pathogenic bacteria; a similar discovery was presented by Liu et al. [89]. BlastKOALA found a series of MFS transporters in the genome of the V2 strain, which may be the reason why V2 has several antibiotic resistance traits. Previous studies have found that the efflux pump mechanism is a common mechanism leading to resistance [90]. When combined with antibiotics, the inherent resistance of lactic acid bacteria may enhance their preventive and therapeutic effects [91]. Overall, strain V2 did not show resistance to Ampicillin, Chloramphenicol and other commonly used antibiotics, and there was no risk of drug resistance gene transfer, which met the safety standards of probiotics.

Whole-genome analysis of *L. plantarum* V2 clarified its genetic characteristics and highlighted its probiotic potential. The GC content of this strain is comparable to that of representative strains such as WFS1 and FCa3L [92,93], with approximately 17.1% of its genes remaining as unannotated sequences, suggesting the need for further functional annotation of the V2 genome. In probiotic systems, the CRISPR feature is beneficial for developing safer and more stable strains: it enhances phage resistance and prevents the spread of plasmids carrying antibiotic resistance genes (ARGs), thereby limiting ARG diffusion and improving strain safety [94]. Prophage-encoded adhesion genes and adaptive genetic elements enhance bacterial stress tolerance, yet prophage-carried virulence genes may augment host pathogenicity, converting non-pathogens to pathogens under specific conditions [95]. In this study, no virulence factors or antibiotic resistance genes were found in the genomic islands or prophage regions of *L. plantarum* V2. Combined with hemolysis assay results, these findings collectively confirm that *L. plantarum* V2 possesses a high safety profile.

Bacteriocins are potent antibacterial substances synthesized by bacteria; lactic acid bacteria are the primary producers of bacteriocins [96,97]. Like other *L. plantarum* strains, the genome of *L. plantarum* V2 contains genes encoding plantaricin F and plantaricin E, indicating its potential to antagonize pathogen colonization and maintain intestinal flora balance [98]. Genome-based functional annotation, combined with predictions of carbohydrate enzyme synthesis genes and metabolic gene clusters, revealed that strain V2 harbors 129 carbohydrate-active enzyme (CAZy) genes and a gene cluster associated with short-chain fatty acid (SCFA) metabolism. Glycoside hydrolases (GHs), key enzymes in carbohydrate metabolism, can degrade complex carbohydrates [99]. Additionally, the presence of glycosyltransferases (GTs) suggests notable probiotic potential, as these enzymes may effectively inhibit pathogenic microorganisms and modulate immune responses [100]. SCFAs, primarily acetic acid, propionic acid, and butyric acid, serve as the main energy source for intestinal epithelial cells and play critical roles in maintaining intestinal barrier integrity, gut homeostasis, and colonic health [101]. These findings indicate that *L. plantarum* V2 exhibits robust metabolic activity, with the potential to significantly contribute to host health through metabolism of host-derived carbohydrates, as well as involvement in pyruvate metabolism and nitrate reduction pathways.

## 5. Conclusions

*L. plantarum* V2 possesses strong tolerance to gastrointestinal-associated stress, high biological safety, moderate epithelial-cell adhesion ability, and broad-spectrum antimicrobial activity against various pathogenic bacteria. Genomic analysis not only further confirmed the favorable safety profile of strain V2, but also uncovered an array of genes linked to probiotic functions, stress-adaptive responses, bacteriocin biosynthesis, production of other bioactive secondary metabolites, and bile-salt tolerance. These superior traits collectively highlight its considerable potential as a candidate strain for feed manufacturing and animal health management. The present study offers a comprehensive assessment of this probiotic strain at both the in vitro and genomic levels. Nevertheless, before practical industrial deployment, further in vivo studies on its functional performance, including immunomodulatory effects and gut microbiota modulation, are still required to fully verify its application prospects.

## Figures and Tables

**Table 1:** Antibacterial activity of strain V2.

Antibiotic	Diameter of Inhibition Zone (mm) Mean ± SE	
	*L. rhamnosus* LGG	*L. plantarum* V2
*Enterotoxigenic Escherichia coli* ETEC K88	15.63 ± 0.23; (++)	14.03 ± 0.13; (++)
*Escherichia coli* ATCC 25922	15.46 ± 0.24; (++)	17.40 ± 0.28; (+++)
*Escherichia coli* (O157:H7) ATCC 43888	16.03 ± 0.40; (+++)	21.71 ± 0.04; (+++)
*Staphylococcus aureus* ATCC 6538	15.68 ± 0.14; (++)	16.84 ± 0.05; (+++)
*Proteus vulgaris* ATCC 29905	18.42 ± 0.21; (+++)	19.88 ± 0.21; (+++)
*Shigella boydii* ATCC 9207	18.94 ± 0.13; (+++)	23.90 ± 0.47; (+++)
*Yersinia enterocolitica* ATCC 23715	21.32 ± 0.31; (+++)	24.13 ± 0.50; (+++)
*Salmonella enterica* ATCC 14028	16.54 ± 0.24; (+++)	13.41 ± 0.15; (++)
*Pseudomonas aeruginosa* ATCC 27853	18.26 ± 0.27; (+++)	19.36 ± 0.17; (+++)

Note: The values are expressed as mm (mean ± SE). 12–16 mm indicates strong positivity (++), and >16 mm indicates extremely strong positivity (+++).

**Table 2:** The inhibitory ability of V2 to *Mycoplasma*.

*Mycoplasma* spp.	Diameter of Inhibition Zone (mm) Mean ± SE	
	*L. casei* V5	*L. plantarum* V2
*Mycoplasma bovis*	13.53 ± 0.23	15.89 ± 0.19
*Mycoplasma capricolum* subsp. *capricolum*	12.36 ± 0.16	15.08 ± 0.08
*Mycoplasma mycoides* subsp. *capri*	-	-
*Mycoplasma agalactiae*	-	-
*Mycoplasma ovipneumoniae*	12.68 ± 0.15	14.74 ± 0.19
*Mycoplasma gallisepticum*	-	-
*Mycoplasma leachii*	12.98 ± 0.38	16.59 ± 0.23

Note: The values are expressed as mm (mean ± SE). “-” indicates no inhibition zone was observed.

**Table 3:** Results of the V2 antibiotic susceptibility test.

Antibiotic	Antibiotic Susceptibility	
	*L. rhamnosus* LGG	*L. plantarum* V2
Ampicillin	S^21.38^	S^25.83^
Kanamycin	R^0.00^	R^0.00^
Gentamicin	R^10.26^	R^9.59^
Chloramphenicol	S^21.27^	S^21.58^
Streptomycin	R^9.87^	R^0.00^
Tetracycline	S^24.15^	I^16.00^
Nalidixic acid	S^21.53^	R^0.00^
Amoxicillin	I^18.79^	S^23.67^
Cefotaxime	I^19.11^	I^17.88^
Erythromycin	S^26.21^	I^18.98^
Vancomycin	R^0.00^	R^0.00^
Clindamycin	S^25.67^	I^18.12^

Note: According to the diameter of the inhibition zone, the zones are divided into drug resistance (R), intermediate resistance (I) and sensitivity (S) zones. The specific reference standard is the CLSI, which is listed in Supplementary Table S1.

**Table 4:** Genomic features of *L. plantarum* V2 and predicted genomic elements.

Attributes	Values
Genome size (bp)	3,261,808
Plasmids	1
GC content (%)	44.48
5S_rRNA	6
16S_rRNA	5
23S_rRNA	5
tRNA	71
Total predicted CDSs	3041
Genomic island	6
Prophage	1
CRISPR number	12
Gene cluster	5
Promoter	826
Genome accession numbers (GenBank)	CP180259–CP180260

**Table 5:** Functional annotations.

Database	Values
Nr	3038
GO	2400
KEGG	1448
eggNOG	2520
Pfam	2516
SwissProt	1686
TrEMBL	3036
All_Annotated	3039

**Table 6:** The annotation results of the whole genome of strain V2 in the proprietary database.

Database	Values
CAZy	129
TCDB	797
CARD	1
VFDB	108
PHI-base	478
Signal peptides	168
Transmembrane protein	865
Secretory proteins	168

## Data Availability

The raw data presented in this study can be found in the NCBI repository with accession number CP180259–CP180260.

## Supplementary Materials

The following supporting information can be downloaded at: https://www.mdpi.com/article/10.3390/microorganisms14102222/s1, Figure S1: ANI analysis of *L. plantarum* V2; Table S1: CLSI antibiotic resistance criteria; Table S2-1: Tolerance to gastric acid; Table S2-2: Tolerance to bile salts; Table S3: Radical scavenging rate (%); Table S4: Adhesion rate (%); Table S5: ANI analysis strain registration number.
