# Supplementary material for: Probiotic Characteristics and Whole-Genome Profiling of Lactiplantibacillus plantarum V2 Isolated from Tibetan Plateau Traditional Yogurt
Source: Microorganisms. 2026 Oct 2;14(10):2222. doi: 10.3390/microorganisms14102222 (PMC13707540; doi:10.3390/microorganisms14102222)
Supplement: Supplementary file 1 [file microorganisms-14-02222-s001.zip › microorganisms-4587574-supplementary.pdf]

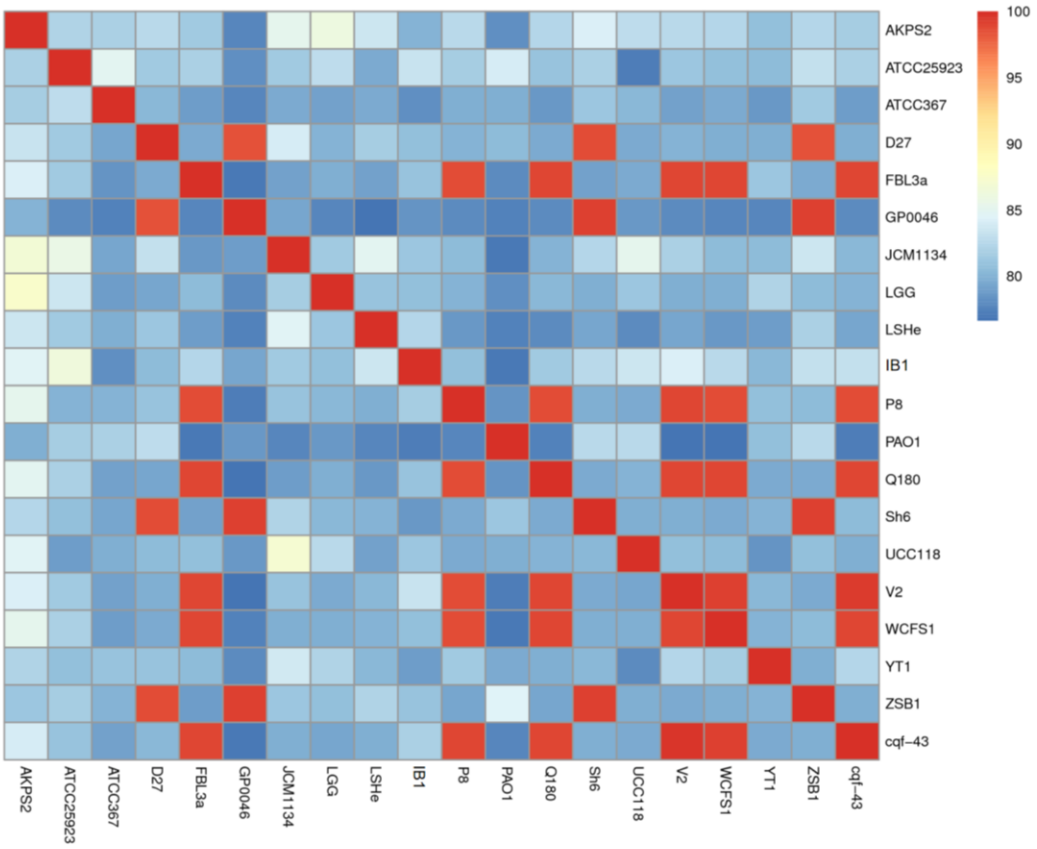

Figure S1. ANI analysis of *L.plantarum* V2

Supplementary Table S1 CLSI antibiotic resistance criteria

Antibiotic category	Antibiotic	The concentration of drug sensitive tablets / (μg · tablet^{-1})	Bacteriostatic circle diameter judgment standard / mm		
			R	I	S
β − lactams	Ampicillin	10	≤13	14~16	≥17
	Amoxicillin	20	≤13	14~17	≥18
	Cefotaxime	30	≤22	23~25	≥26
Macrolides	Erythromycin	15	≤13	14~22	≥23
Tetracycline	Tetracycline	30	≤11	12~14	≥15
Chloramphenicol	Chloramphenicol	30	≤12	13~17	≥18
Aminoglycosides	Kanamycin	30	≤13	14~17	≥18
	Streptomycin	10	≤11	12~14	≥15
	Gentamicin	10	≤12	13~14	≥15

Antibiotic category	Antibiotic	The concentration of drug sensitive tablets / (μg · tablet^{-1})	Bacteriostatic circle diameter judgment standard / mm		
			R	I	S
Glycopeptide	Vancomycin	30	–	–	≥15
Quinolone	Nalidixic acid	30	≤13	14~18	≥19
Lincosamides	Clindamycin	2	≤14	15~20	≥20

Note：R: drug resistance, I: neutral sensitivity, S: sensitivity, -: no inhibition zone, The same as follows.

Supplementary Table S2-1 Tolerance to gastric acid

bacterial survival rate（%）	
L．*rhamnosus*LGG	*L*．*plantarum*V2
72.12	83.31
72.57	85.71
73.02	80.05

Supplementary Table S2-2 Tolerance to bile salts

Bacterial growth rate(%)	
L．*rhamnosus*LGG	*L*．*plantarum*V2
17.83	19.68
19.02	21.28
18.82	20.07

Supplementary Table S3 Radical scavenging rate (%)

	Radical scavenging rate (%)	
	**DPPH**	**ATBS**
	59.68	78.55
***L．plantarum*V2**	58.91	81.68
	61.13	82.66

Supplementary Table S4 Adhesion rate (%）

Adhesion rate (%）	
***L*．*rhamnosus* LGG**	***L*．*plantarum*V2**
7.33	1.88
8.02	1.72
8.71	1.88

Supplementary Table S5 ANI analysis strain registration number

**strain**	**NCBI accession number**
Bacillus subtilis strain AKPS2	NZ_CP172417.1
Lactobacillus brevis strain ATCC 367	CP000416.1
Lactiplantibacillus plantarum strain cqf-43	CP139651.1
Staphylococcus aureus strain ATCC 25923	NZ_CP009361.1
Ligilactobacillus animalis strain D27	NZ_CP173167.1
Lactiplantibacillus plantarum strain FBL-3a	CP034694.1
Ligilactobacillus animalis strain GP0046	NZ_JAUNZL010000010.1
Lacticaseibacillus paracasei strain IB1	CP162491.1
Lactiplantibacillus plantarum strain LB34	MT994869.1
Lacticaseibacillus rhamnosus strain *GG*	NC_013198.1

**strain**	**NCBI accession number**
Limosilactobacillus reuteri strain LSHe	CP170358.1
Pseudomonas aeruginosa strain PAO1	AE004091.2
Lactobacillus plantarum subsp. plantarum strain P-8	CP005942.2
Lactiplantibacillus plantarum strain Q180	NZ_CP073753.1
Lactobacillus salivarius strain UCC118	CP000233.1
Lactiplantibacillus plantarum strain WCFS1	NC_004567.2
Ligilactobacillus animalis strain ZSB1	NZ_CP123751.1
*Lactobacillus acidophilu*s strain YT1	NZ_CP025200.1
Lactobacillus animalis strain Sh6	JBXUMF000000000.1
